# Investigating the Potential of Ghee Precursor-Derived Carbon Nano Onions for Enhancing Interfacial Bonding in Thermoplastic Composites

**DOI:** 10.3390/molecules29050928

**Published:** 2024-02-20

**Authors:** Kailashbalan Periasamy, Maryam Darouie, Raj Das, Akbar A. Khatibi

**Affiliations:** School of Engineering, RMIT University, Melbourne, VIC 3000, Australiamaryam.darouie@rmit.edu.au (M.D.); raj.das@rmit.edu.au (R.D.)

**Keywords:** carbon soot, carbon nano onions, interfacial bonding, carbon fiber, PA6

## Abstract

In this study, we employed a straightforward flame synthesis process to produce carbon soot containing carbon nano onions (CNOs) using easily accessible ghee oil as a precursor. The ghee oil, with a molecular composition rich in more than 50 carbon atoms, served as an effective source for generating CNOs. The synthesized CNO particles underwent comprehensive characterization through high-resolution transmission electron microscopy (HRTEM), energy dispersive X-ray spectroscopy (EDX), Fourier transform infrared spectroscopy (FTIR), and X-ray diffraction (XRD) analyses, providing a detailed account of their physicochemical properties. In addition, we explored the direct deposition of CNOs on carbon fiber (CF) surfaces for 5 and 10 min via a soot deposition process. The resulting freeze–fracture images obtained from scanning electron microscope (SEM) offered insights into the morphology of the CNO-deposited CF. Our study aims to shed light on the potential applications of CNOs, focusing on their characterization and the possible benefits they may offer in diverse fields, including but not limited to enhancing interfacial bonding in thermoplastic composites.

## 1. Introduction

Carbon fiber-reinforced plastics (CFRP) have become indispensable in a wide array of industries, including aerospace, automotive, and marine applications, primarily due to their exceptional properties, such as high specific strength, corrosion resistance, and lightweight nature. The aerospace sector leverages CFRP for its ability to reduce overall weight without compromising structural integrity, leading to enhanced fuel efficiency and performance. In the automotive industry, CFRP components contribute to improved fuel economy and reduced emissions, aligning with the growing emphasis on sustainability and environmental impact. Similarly, in marine applications, CFRP offers advantages such as high stiffness and resistance to saltwater corrosion. Within the realm of CFRP composites, the thermoplastic variants hold a distinguished position due to their unique attributes, including weldability and recyclability [1].

Despite these advantages, the challenge lies in the weakened interfacial bond between the carbon fibers and thermoplastic polymers during solidification. This compromised bonding can lead to premature failure of the composite structure. As industries continue to seek lightweight and durable materials, addressing the interfacial bonding issues in thermoplastic composites becomes paramount. Enhancing this bond is crucial not only for structural integrity but also for unlocking the full potential of thermoplastic composites in various applications. Improved interfacial bonding can result in composites with higher strength, increased resistance to fatigue, and better overall performance, making them even more appealing for use in critical components across diverse industrial sectors. Hence, research endeavors focusing on innovative solutions to reinforce the fiber–matrix interface in thermoplastic composites are imperative for advancing the widespread adoption of these materials in different industrial contexts.

To surmount the challenge of weakened interfacial bonding in thermoplastic composites, a common strategy involves the utilization of nano/microparticles to modify the fiber surface, thereby enhancing its connection with thermoplastic matrices. This modification primarily operates through mechanical interlocking mechanisms, reinforcing the overall composite structure. Carbon-based nanoparticles, including carbon nanotubes (CNT) [2], graphene nanoplatelets (GNP) [3], graphene oxide [4], and reduced graphene oxide [5], emerge as particularly promising candidates for such applications. Their resilience to high temperatures and pressures during the hot-pressing process makes them well suited for integration into thermoplastic composites. The use of these carbon-based nanoparticles aims not only to improve interfacial bonding but also to impart additional functionalities, such as enhanced mechanical properties and thermal conductivity, further expanding the applicability of thermoplastic composites in various industrial settings. As industries increasingly demand materials with superior performance and multifunctionality, the exploration and optimization of nano/microparticle modifications represent crucial steps toward advancing the capabilities and versatility of thermoplastic composites in diverse applications.

While one-dimensional nanoparticles like CNTs with high aspect ratios can augment mechanical interlocking, their cost and impracticality for large-scale composite production limit their utility. Two-dimensional nanoparticles, like GNPs, effectively improve fiber–matrix adhesion but are susceptible to agglomeration and are less cost effective for continuous production. Recently, zero-dimensional nanoparticles such as carbon black nano powders have been utilized to strengthen fiber/matrix bonding in both thermoset [6,7,8,9,10] and thermoplastic [11,12] composites. Surprisingly, carbon nano onions (CNOs) derived from ghee lamp combustion have not been explored for interfacial engineering. The concentric graphite layers and porous structure of CNOs [13] suggest their potential to absorb molten thermoplastic during hot pressing, thereby enhancing interfacial strength beyond baseline samples. It is hypothesized that CNOs can contribute to crack retardation through toughening mechanisms like crack deflection, fiber bridging, particle rupture, and particle pull-out.

Initially reported as by-products during carbon nanotube synthesis by Lijima in 1980 [14], various techniques such as electric arc discharge [15], laser ablation [16], and electron beam irradiation [17] have been employed to synthesize CNOs. However, these methods come with drawbacks, including catalyst contamination, the need for sophisticated instruments, and high vacuum working conditions. Moreover, the limited production of CNOs from these techniques may not suffice for large-scale composite fabrication. To address these challenges, a recently developed simple flame synthesis process, utilizing readily available ghee oil [13], offers a viable solution for continuous CNO production, paving the way for their integration into carbon fiber-reinforced thermoplastic composites (CFRTPs).

This study provides a comprehensive characterization of carbon nano onions derived from a ghee precursor, employing high-resolution transmission electron microscopy (HRTEM), energy dispersive X-ray spectroscopy (EDX), Fourier transform infrared spectroscopy (FTIR), and X-ray diffraction (XRD) analyses. To investigate the practical application of these CNOs, the carbon fiber (CF) surface undergoes a soot deposition process, effectively coating it with CNOs. The modified fibers, in conjunction with a polyamide 6 (PA6) matrix, are then subjected to a hot-pressing process to fabricate composite samples. Subsequent to the mechanical testing phase, the samples undergo freeze fracture, facilitating scanning electron microscope (SEM) analysis to delve into the intricate details of the fracture morphology. This multi-faceted characterization approach provides a holistic understanding of the CNOs and their influence on the composite structure, laying the foundation for advancements in interfacial engineering for carbon fiber-reinforced thermoplastic composites.

## 2. Exploring the Effect of Carbon Soot Deposition on Fiber–Matrix Bonding

**Figure 1:**
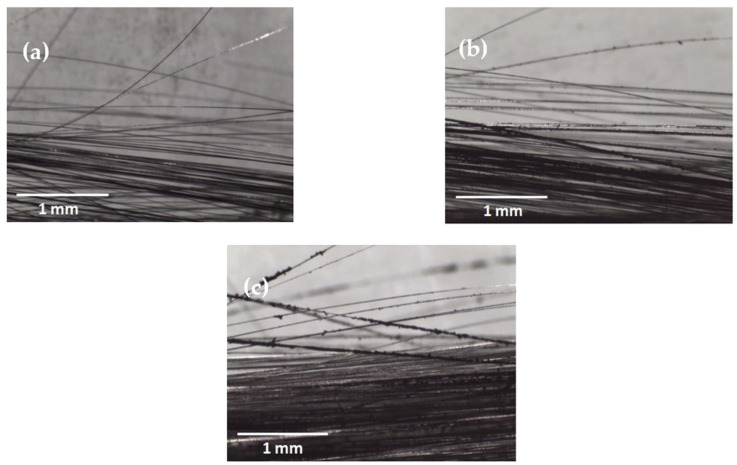
Optical microscope images of (**a**) untreated CF; CFs with carbon soot deposited for (**b**) 5 min and (**c**) 10 min.

The soot particles were deposited layer by layer on the carbon fiber (CF) tow, and the quantity of carbon soot increased with prolonged process duration, as illustrated in Figure 1. The untreated CFs, originally smooth, became visibly darker and rougher as more soot settled on their surface. Carbon soot, carrying agglomerated carbon nano onions, adhered to the fibers through electrostatic absorption. This Van der Waals interaction potentially forms non-covalent chemical bonds between the CNOs in soot and the CF surface [18]. However, the CNOs were deposited in an agglomerated state during the soot deposition process rather than in a well-dispersed form. This agglomeration weakened the adhesive strength between the CF surface and CNO particles, resulting in potential CNO detachment, particularly noticeable in the 10-minute processed CFs carrying larger soot particles. Despite the loose bonding, the deposited CNOs could enhance the fiber surface roughness, facilitating increased interlocking with the polyamide 6 (PA6) matrix. Additionally, the functional groups on CNO particles could contribute to improved interfacial bonding between the PA6 matrix and CNOs. A preliminary study explored the possible contribution of deposited CNOs to interfacial bonding in thermoplastic composites.

Lap joint samples were meticulously crafted by stacking two soot-deposited CF tows, with a PA6 matrix sandwiched in between, and subjected to hot pressing at 230 °C for a duration of 20 min. In parallel, corresponding lap joint samples were prepared utilizing untreated CFs, providing crucial baseline references for comparative analysis. The distinct samples were systematically labeled as UBL, 5MCD, and 10MCD, representing untreated CFs, 5 min of soot-modified CFs, and 10 min of soot-modified CFs, respectively. Rigorous lap shear tests were executed on the samples until failure, followed by a meticulous lateral breakage of the fractured samples after cryogenic freezing with liquid nitrogen, providing a comprehensive cross-sectional view to glean insights into the interfacial characteristics and the potential effects of varying soot deposition durations on fiber–matrix bonding.

**Figure 2:**
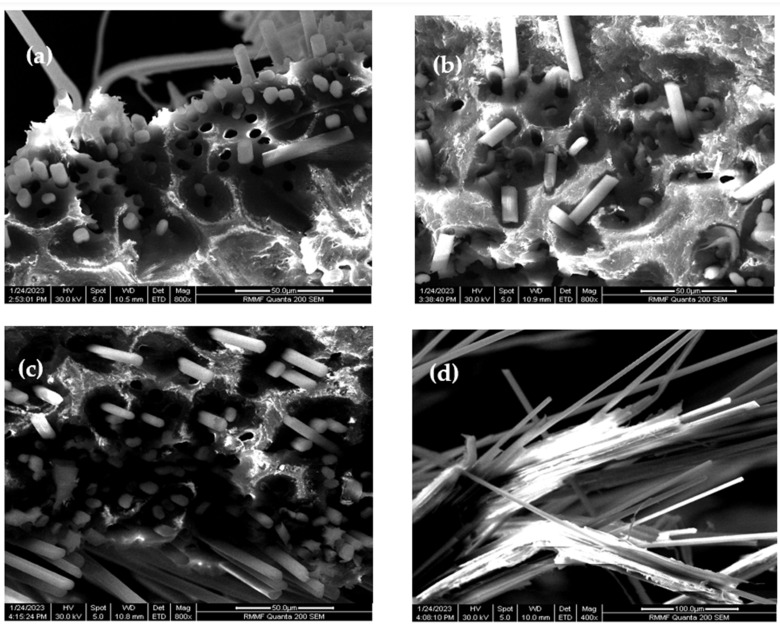
SEM images of CF/PA6 fracture morphology: (**a**) UBL sample, (**b**) 10MCD sample, (**c**) 5MCD sample, and (**d**) PA6 matrix attached to the broken fibers of 5MCD samples.

Inspection of SEM images depicting the CF/PA6 fracture morphology (Figure 2a–d) underscores a consistent occurrence of fiber breakages and voids attributed to fiber pull-outs across all samples. Significantly, the 5MCD (Figure 2c,d) samples stand out with a notable reduction in voids resulting from pulled-out CFs when compared to both the UBL (Figure 2a) and 10MCD (Figure 2b) samples. This observed trend implies a potential enhancement in fiber–matrix interfacial bonding facilitated by the deposition of 1 mg of carbon soot during the 5-min process. The discernible presence of the PA6 matrix firmly attached to the fractured fibers in the 5MCD samples further bolsters the evidence for improved fiber–matrix adhesion, offering valuable insights into the impact of varied soot deposition durations on the interfacial characteristics of the composite material.

**Figure 3:**
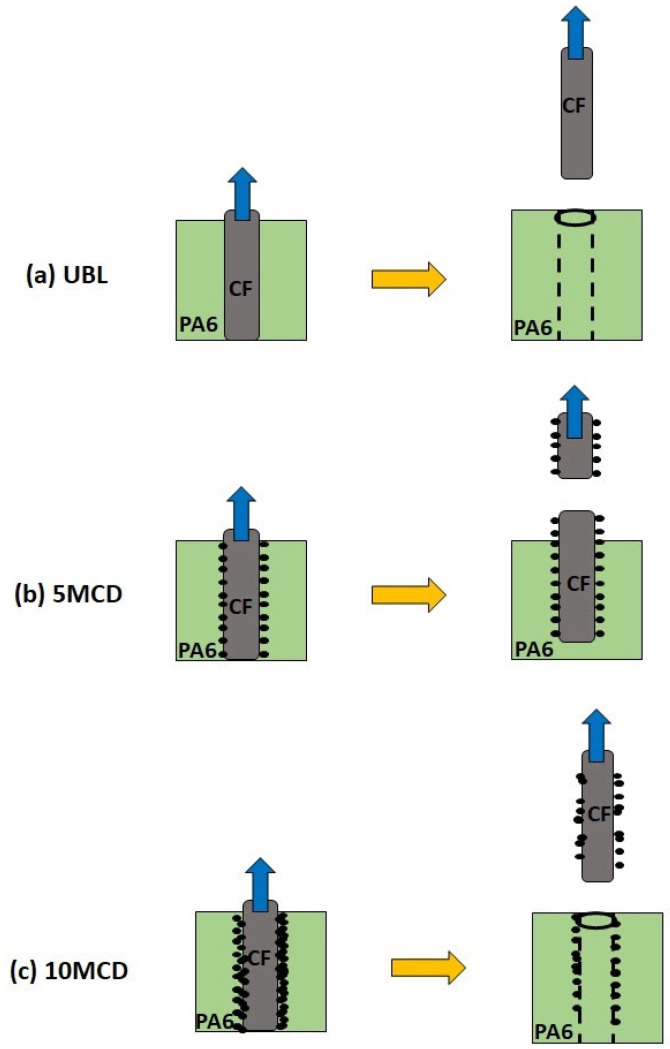
Schematic illustrating the interfacial fracture mechanism of CF/PA6-CNO composites with varying process durations. The figures on the left depict specimens immediately after load application, while those on the right represent the situation after the final rupture.

Based on the fracture morphology results, a schematic of the possible interfacial fracture mechanism for CF/PA6-CNO samples is presented in Figure 3. For UBL samples, the smooth untreated CF surface lacked strong bonding with the PA6 matrix, resulting in extensive fiber pull-outs and void formation. After depositing soot particles carrying CNOs for 5 min, the coated particles facilitated mechanical interlocking between CFs and the PA6 matrix, strengthening the CF filaments’ bond to the PA6 matrix. This led to filament breakage upon surpassing the interfacial strength between CFs and PA6. In the case of 10MCD samples, although voids due to fiber pull-out were slightly lower than in UBL samples, more fibers experienced pull-out due to excessive soot particles, leading to increased stress concentration at the interface and reduced bonding. This suggests that the presence of soot particles at the interfacial region can enhance fiber–matrix bonding to some extent.

Importantly, it must be emphasized that the discussion remains qualitative, and the absence of quantitative data on fiber–matrix bonding underscores the need for prudence in drawing definitive conclusions regarding the magnitude of bonding enhancement. While the results offer valuable qualitative insights into the interfacial bonding mechanism, there is a clear recognition of the necessity for subsequent quantitative investigations to provide a more comprehensive understanding of the observed effects and to further substantiate the qualitative findings presented in this study.

## 3. Preparation and Characterization of CNO Particles

### 3.1. Flame Synthesis of CNOs

**Figure 4:**
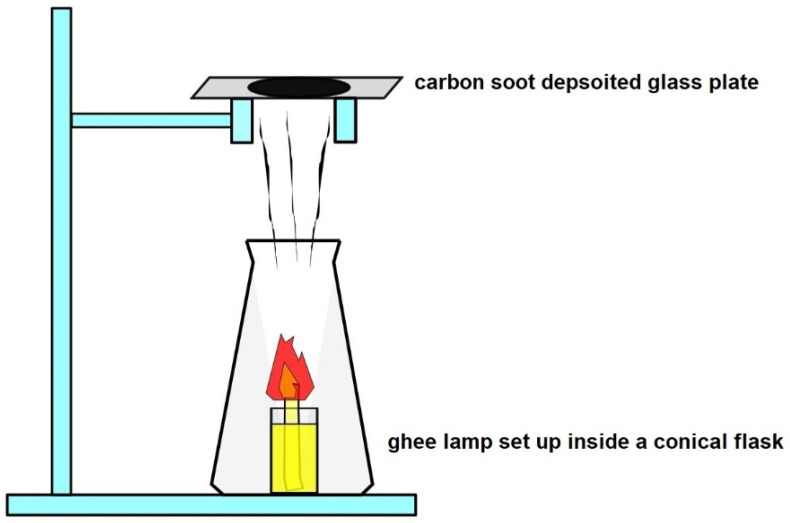
Schematic diagram of the flame synthesis method for producing CNOs using ghee oil—The ghee lamp is strategically placed inside a conical flask to manage carbon smoke dispersion, directing it towards the glass collector plate.

The synthesis of carbon nano onions was accomplished through a straightforward flame synthesis method [13], utilizing ghee, commonly known as clarified butter, as the precursor material. The synthesis procedure involved the systematic filling of a small beaker with ghee oil, into which a cotton wick was immersed. The exposed end of the wick was ignited to initiate a glowing ghee lamp. To ensure controlled dispersion of carbon smoke and its directed deposition onto the glass collector plate, the ghee lamp was strategically placed inside a conical flask, as illustrated in Figure 4. The resulting carbon smoke emitted by the ghee lamp underwent condensation, forming a uniform coating of carbon soot on the glass plate. This distinctive layer was meticulously scraped from the plate using a spatula, setting the stage for subsequent analysis and material characterization. This flame synthesis approach using ghee as the precursor material not only exemplifies simplicity but also introduces a novel and accessible method for the continuous production of CNOs, showcasing its potential significance in the realm of carbon nanomaterial synthesis. The method employed to transfer CNOs into the silicon substrates involved mixing carbon soot containing carbon nano onion particles with isopropanol followed by sonication for 1 h, and subsequently transferring the CNO solution to a Si substrate.

### 3.2. Characterization of CNO Particles

In this study, the characterization of carbon nano onions was conducted to confirm the presence of concentric graphite layers, which have the potential to enhance bonding between the fibers and matrix through capillary action. The primary objective of this manuscript is to demonstrate the viability of CNOs derived from ghee in the fabrication of thermoplastic composite structures. It should be noted that the full characterization of ghee-derived CNOs is beyond the scope of this paper. Detailed characterization of ghee-derived CNOs has been previously reported in studies conducted by Mohapatra et al. [13] and Mongwe et al. [19].

#### 3.2.1. HRTEM Results on the Morphology of CNOs

**Figure 5:**
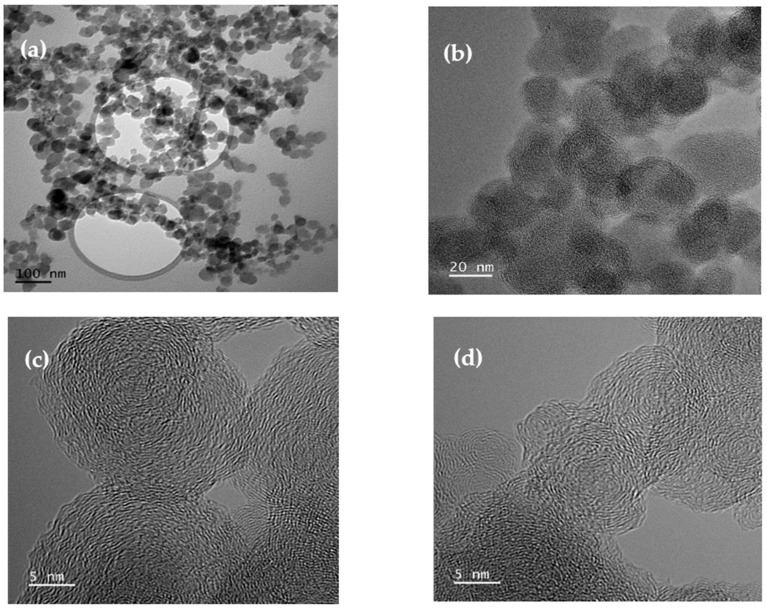
(**a**) TEM image showing the network structure of CNOs, and HRTEM images showing (**b**) overlapping CNOs, (**c**) distinguishable cores of CNO, and (**d**) interpenetrating graphite planes.

The morphological analysis of carbon nano onions was meticulously conducted using the F200 high-resolution transmission electron microscopy (HRTEM) from JEOL (Tokyo, Japan), presenting the obtained images in Figure 5. The CNOs exhibited a distinctive spherical morphology with diameters spanning from 20 to 50 nm, collectively forming an intricate network structure. Noteworthy observations included concentric graphite layers featuring multiple nucleation centers within the CNOs. Figure 5a offers a comprehensive TEM image, providing an overview of the network structure. Additionally, Figure 5b–d present HRTEM images that delve into specific features, including overlapping CNOs (Figure 5b), distinguishable cores of CNOs (Figure 5c), and interpenetrating graphite planes (Figure 5d). This morphological analysis offers a detailed insight into the structural intricacies of CNOs, laying the groundwork for a comprehensive understanding of their potential applications.

**Figure 6:**
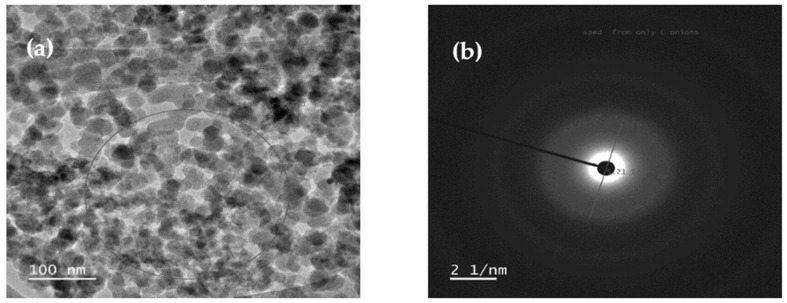
HRTEM images displaying (**a**) the circular region selected for diffraction pattern and (**b**) the resulting SAED pattern for CNOs.

The crystallographic arrangement of the synthesized carbon nano onions was further elucidated through selected area electron diffraction (SAED) images, as showcased in Figure 6. The analysis revealed an interlayer distance of approximately 0.38 nm, signifying the presence of defects in the graphitic carbon [13]. Figure 6 provides valuable insights into the crystallographic characteristics of the CNOs, enhancing our understanding of their structural properties. The determination of the interlayer distance, a critical parameter in graphitic carbon, adds significant depth to the characterization of CNOs. This comprehensive analysis, coupling HRTEM with SAED, not only enriches the morphological understanding of CNOs but also contributes crucial information, including defect analysis; stacking arrangement, crystallinity, and size and phase identification, for evaluating their potential in diverse applications, especially as secondary reinforcements in thermoplastic composites. Note that in this study, SAED pattern analysis was employed solely to investigate the interlayer distance of the graphitic planes in the prepared carbon nano onions and to ascertain the presence of defects. Consequently, detailed information, such as the crystal planes of each ring was not obtained.

The growth mechanism elucidating the formation of solid carbon nano onions from ghee oil underscores the intricate composition of saturated and unsaturated fats. This process unfolds during the flame synthesis, reaching temperatures of nearly 1000 °C, where these fats undergo combustion, resulting in the breakdown into carbon radicals. These radicals subsequently engage in nucleation and growth processes, culminating in the creation of quasi-spherically shaped carbon nanomaterial with concentric multilayers [19]. The size of the CNOs is intricately regulated by the nucleation and accretion phenomena inherent in the carbon radicals generated within the flame. The physicochemical properties and yield of the CNOs emerge as outcomes influenced by a myriad of parameters, encompassing the quantity of ghee oil utilized, the duration of the synthesis process, the spatial relationship between the lamp and collector plate, the characteristics of the wick including type and size, and the thermal conductivity of the collector plate integral to the soot deposition process [19]. This comprehensive growth mechanism not only provides a detailed account of the intricate processes governing CNO formation but also offers valuable insights for the optimization of CNO synthesis, paving the way for their controlled application in various composite structures.

The growth mechanism described above offers valuable insights for optimizing carbon nano onion synthesis in several ways. Firstly, by delineating the parameters affecting CNO synthesis, such as ghee oil quantity and synthesis duration, the mechanism provides clarity on the critical factors influencing CNO formation. This understanding of key factors transitions seamlessly into the ability to manipulate synthesis conditions, gained through insight into nucleation and growth processes, allowing researchers to control the size, morphology, and yield of CNOs, thereby facilitating their tailored production for specific applications. Moreover, understanding the relationship between synthesis parameters and CNO properties enables optimization efforts to focus on maximizing yield and enhancing the efficiency of the synthesis process, contributing to efficiency enhancement. Ultimately, the optimized synthesis of CNOs creates opportunities for their controlled integration into various composite structures, ensuring that their properties align with the requirements of specific applications, thereby enabling their application in composite structures.

#### 3.2.2. EDX Results on the Chemical Composition of CNOs

**Figure 7:**
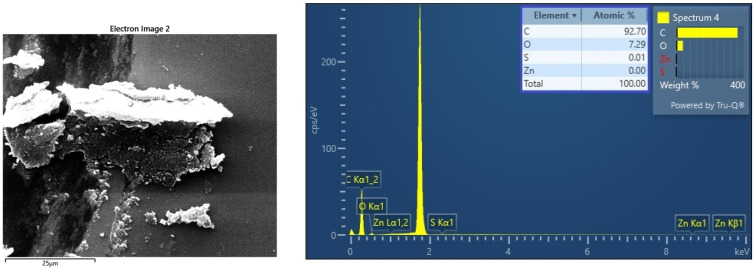
EDX spectrum of flame-synthesized CNOs, highlighting the predominant carbon and oxygen composition.

Energy dispersive spectroscopy (EDX) analysis was meticulously conducted using the FEI Quanta 200 scanning electron microscope (SEM), offering valuable insights into the elemental composition of the lab-prepared carbon nano onions. The resulting EDX spectrum, presented in Figure 7, serves as a comprehensive illustration of the elemental constituents within the CNO sample. The spectrum vividly highlights the predominant presence of carbon and oxygen, attesting to the high degree of purity achieved in the synthesized CNOs. In particular, the elemental analysis indicates that the CNOs are composed of approximately 93% carbon and 7% oxygen. The resilience of carbonaceous nanomaterials to high temperatures and pressure generated during the hot pressing of thermoplastic composites renders them advantageous. Consequently, a higher carbon content in CNOs implies reduced damage to nanoparticles during hot-pressing processes. This EDX analysis, coupled with the visual representation in Figure 4, adds a layer of precision to the characterization of CNOs, laying the groundwork for a nuanced understanding of their chemical composition, essential for elucidating their potential applications in composite structures.

The EDX analysis carried significant importance, operating under a high-beam mode at 30 kV within the confines of the RMIT Microscopy and Microanalysis Facility (RMMF). A critical aspect of this analysis involved the identification and subsequent management of excessive “Si” signals originating from the silicon substrate. It is imperative to highlight that these signals, while detected by the EDX system, were acknowledged as potential sources of interference in the spectrum analysis. To maintain the integrity and accuracy of the overall assessment, a meticulous approach was adopted to eliminate these silicon signals from the EDX spectrum. This procedural precision ensures that the subsequent interpretation and conclusions drawn from the EDX analysis remain focused and unencumbered by extraneous signals, contributing to the robustness of the characterization of carbon nano onions.

#### 3.2.3. FTIR Results on Functional Groups Present in CNOs

**Figure 8:**
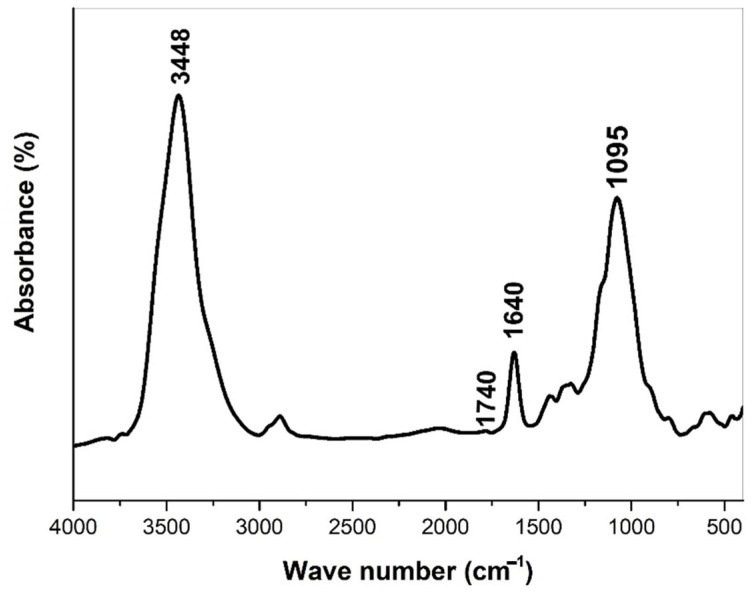
FTIR spectrum illustrating functional groups present in CNOs.

The exploration of functional groups within the prepared carbon nano onions was conducted through Fourier transform infrared spectroscopy (FTIR) analysis employing a KBr pallet, showcased in Figure 8. The resulting FTIR spectrum emerges as a valuable tool for identifying specific functional groups present in the CNOs. Notably, the spectrum delineates the discernible presence of hydroxyl, carboxyl, and carbonyl groups within the intricate structure of the CNOs. This analytical approach enhances our understanding of the chemical composition of CNOs, shedding light on the diverse functional moieties that contribute to their potential applications in composite materials.

The FTIR spectrum provides a nuanced glimpse into the functional groups within the prepared carbon nano onions, revealing distinctive peaks that contribute to the complex molecular composition. A notable broad peak at 3448 cm^−1^ signifies the O-H vibrational stretch of the carboxylic group, underscoring the presence of oxygen-containing functional entities. Accompanying this is a shoulder observed at 1740 cm^−1^, which provides clear evidence of the C=O vibrational stretch associated with the carboxylic group, thus adding a layer of specificity to the functional groups identified. Additionally, the discernible peak at 1640 cm^−1^ emerges as a distinct marker, attributed to the C=C stretch of the alkene group, showcasing the diverse carbon-based constituents within the CNO structure. Another noteworthy peak at 1095 cm^−1^ corresponds to the C-C stretch of the aromatic rings, further enriching the spectral landscape and emphasizing the intricate nature of the carbon nano onions. This detailed analysis of the FTIR spectrum enhances our understanding of the specific functional groups embedded within the CNOs, laying a foundation for a comprehensive exploration of their potential applications in composite materials.

The development of hydroxyl and carboxyl groups within carbon nano onions finds its origin in the combustion process of fats present in ghee oil. This intricate transformation takes place within the ghee lamp setup, reaching temperatures close to 1000 °C. Notably, this distinctive feature sets CNOs apart from many other nanoparticles, as their synthesis inherently incorporates oxygen-based functional groups. Unlike counterparts that often require post-synthesis treatments involving concentrated acids to introduce functional moieties and improve dispersion, CNOs emerge from their synthesis with pre-existing oxygen-based functional groups. This inherent characteristic negates the need for additional post-processing with harsh chemicals, thereby preserving the morphology of CNOs. This unique attribute not only sustains their effectiveness in enhancing interfacial bonding but also ensures the maintenance of their structural integrity, contributing to their potential as versatile agents in composite materials.

#### 3.2.4. XRD Results on the Crystallinity of CNOs

**Figure 9:**
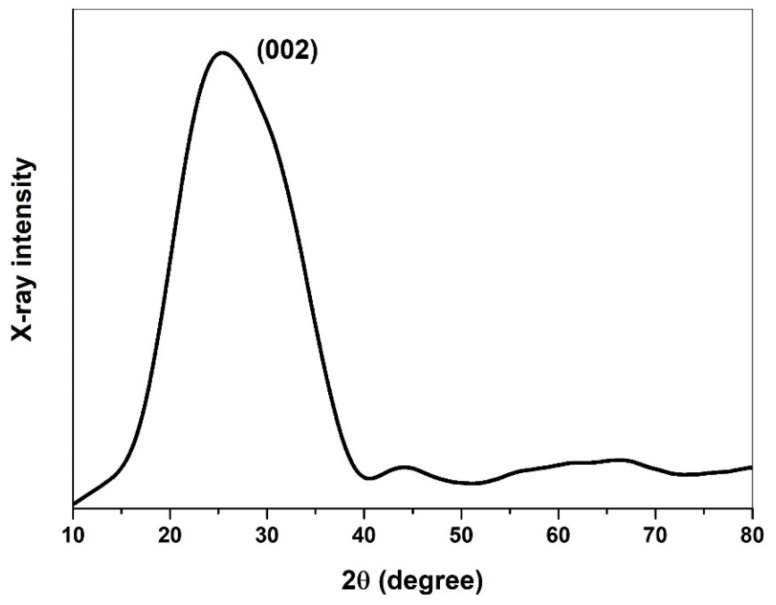
X-ray diffraction of the prepared CNOs, highlighting the graphitic carbon peak.

The powder X-ray diffractometer supplied by Bruker D4 Endeavor was used in our work to study the X-ray diffraction (XRD) spectra of CNO particles. The XRD analysis of the synthesized CNOs, as depicted in Figure 9, unveils a prominent graphitic peak at a 2θ angle of 25 to 26°, signifying the ordered arrangement of graphite carbon in the (002) direction. The breadth of this (002) graphite peak indicates the amorphous nature of the prepared CNOs and suggests the presence of graphite layers with a short domain order. The position of the (002) peak was scrutinized, revealing an interlayer distance (d002) of 3.55 Å, slightly higher than the interlayer distance of 3.36 Å observed in well-crystallized graphite. This discrepancy points to the existence of defects in the graphitic carbon of CNOs, as corroborated by the HRTEM results. 

Analyzing the (002) peak position, the interlayer distance (d002) was determined to be 3.55 Å, surpassing the 3.36 Å interlayer distance in well-crystallized graphite. This finding aligns with observations in the HRTEM results, confirming the presence of defects in the graphitic carbon of CNOs. To ascertain the crystallite size (*L_c_*), the William–Hall formula was applied as follows:

(1) $$L_{c}=\frac{K\;\lambda }{FWHM\cos\theta -4\varepsilon \;sin\theta }$$

where *L_c_* is the crystallite size, *K* is the shape factor with a value of 0.9, *λ* is the wavelength of incident X-ray (1.5406 Å), *FWHM* is the full width at half of the maximum peak from the (002) graphite carbon, and *θ* is the angle of incident X-ray. The lattice strain (*ε* = 0.057) was determined using the formula (d002 − d0)/d0, where (d002) is the interlayer distance found in the prepared CNOs, and (d0) is the interlayer distance in well-crystallized bulk graphite. Substituting the respective values into the William–Hall formula, the crystallite size (*L_c_*) was found to be 6.14 Å. Consequently, for the observed d-spacing value of 3.55 Å in CNOs, the number of graphite layers present in each crystallite can be estimated to be 1 or 2. The large interlayer spacing (d002) coupled with a low crystallite size (*L_c_*) suggests a small number of graphite layers per crystallite, as indicated by the calculation. The d-spacing and (*L_c_*) values of ghee-derived CNOs in our study align with XRD results obtained for CNOs produced through laser pyrolysis of anthracene [20]. However, enhancing the crystallinity of CNOs synthesized from ghee is achievable by placing the collector plate closer to the flame [19]. In this study, the collector plate was positioned 14 cm from the wick tip, aligning with the working height of the carbon fiber (CF) tow in the soot deposition process. This placement was chosen to prevent potential damage to the CF tow due to the heat generated by the flame.

## 4. CNO Deposition on Carbon Fiber Using a Soot Deposition Process

Various techniques have been employed to introduce nanoparticles at the fiber–matrix interface, encompassing nano-sizing [4], spray coating [21], chemical vapor deposition (CVD) [22], electrophoretic deposition (EPD) [23], and nano grafting using chemicals [24]. However, methods like CVD, EPD, and nano grafting, while effective, present intricacies, time-consuming procedures, challenges in scaling up for large fiber areas, and often involve the use of toxic chemicals. Furthermore, chemical-based techniques demonstrate reduced efficacy in carbon fiber-reinforced thermoplastic composites (CFRTPs) due to the limited capacity of thermoplastics to form covalent chemical bonds with treated fibers or nanoparticles. To surmount the challenges associated with nano inclusion methods, this study introduces a straightforward fiber-modification technique employing CNOs generated from ghee, offering a simpler, scalable, and environmentally friendly alternative.

**Figure 10:**
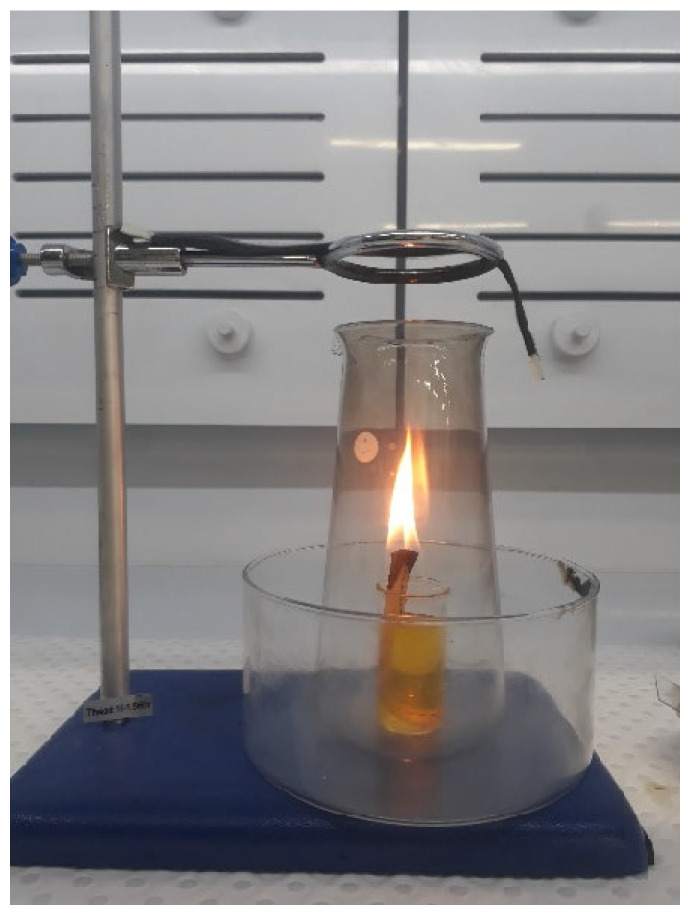
Illustration of the carbon soot deposition process on untreated CF tow. This modification process offers a simplified and efficient alternative to intricate methods for incorporating CNOs onto carbon fibers, addressing the challenges associated with complex and chemical-intensive techniques commonly used in the field. The presented approach allows for uniform CNO coverage on the CF surface, paving the way for enhanced interfacial interactions in CFRTPs.

Following the same steps outlined in the carbon nano-onion (CNO) synthesis process, the ghee lamp setup was prepared. Carbon smoke emitted from the ghee lamp was directly deposited onto the untreated surface of carbon fibers (CFs) for two durations, namely 5 and 10 min, as depicted in Figure 10. Subsequently, after completing the deposition on one side of the CF tow, the tow was inverted, exposing the other side to soot for an additional 5 or 10 min to ensure uniform coverage of CNOs around the fiber surface. The carbon fibers with deposited nanoparticles were then impregnated with PA6 using the hot-press technique. In this study, the weight of carbon nano onions deposited on the 4 cm^2^ surface area of the 24k carbon fiber (CF) tow was determined utilizing the weight difference method. It was noted that CF tow, with soot deposited for 5 and 10 min on both sides, accumulated approximately 1 and 2 mg of carbon soot, respectively. However, it is important to acknowledge that the values presented here represent averages derived from five measurements, each exhibiting minimal variation. Exploring the events occurring between 5 to 10 min of soot particle deposition and whether void reduction intensifies within this timeframe pose intriguing questions. While this study focuses solely on investigating the potential of depositing soot on fiber surfaces and its consequential impact on bonding with a PA6 matrix, a more comprehensive quantitative analysis of the effect of different soot deposition durations on the fiber–matrix interface will be addressed in future research endeavors. Nonetheless, preliminary observations indicate distinct differences in the accumulation of nanoparticles on the carbon fiber surface after 10 min compared to 5 min of exposure, thus anticipating various distributions of CNOs that influence interfacial bonding when incorporated with thermoplastic polymers.

## 5. Conclusions

The carbon nano onions (CNOs) synthesized from ghee oil as a catalyst precursor underwent comprehensive characterization through HRTEM, EDX, FTIR, and XRD analyses. The obtained results, showcasing concentric graphite layers with 93% carbon content and inherent oxygen-based functional groups, underscore the potential of these CNOs as secondary reinforcements in thermoplastic composites. In this study, a straightforward soot deposition process was employed to introduce CNOs to the carbon fiber (CF) surface, investigating their potential impact at the fiber–matrix interface in CF-PA6 samples.

The loaded samples, analyzed through freeze fracture, revealed that the proposed interfacial engineering technique could enhance bonding between the CF and the PA6 matrix, facilitated by the interlocking effect provided by CNOs present in the soot. This qualitative observation suggests that CNOs harbor the potential to augment the interfacial strength of composite materials. The findings encourage further exploration of CNOs in composite structures, paving the way for their optimized application in composite manufacturing. In summary, the synthesized CNOs exhibit promise as effective additives for improving interfacial bonding in thermoplastic composites. The observed enhancements in bonding through the proposed soot deposition process warrant continued investigation and optimization for practical applications in composite manufacturing. This study contributes to the growing body of research on CNOs in composite materials, emphasizing their potential role in advancing the performance of composite structures.

## Data Availability

Data are contained within the article.
